# Epidemiological manifestations of hepatitis C virus genotypes and its association with potential risk factors among Libyan patients

**DOI:** 10.1186/1743-422X-7-317

**Published:** 2010-11-13

**Authors:** Hana A Elasifer, Yossif M Agnnyia, Basher A Al-Alagi, Mohamed A Daw

**Affiliations:** 1 Department of Medical Microbiology & Immunology, Faculty of Medicine and Department of Infectious Diseases Tripoli Medical Centre, Tripoli-Libya

## Abstract

**Background:**

The information on hepatitis C virus genotypes and subtypes among Libyan population and its association with various risk factors is not known. The objectives of this study were to determine the epidemiological manifestations of HCV genotypes among Libyan patients and their association with certain potential risk factors.

**Methods:**

A total of 1240 of HCV infected patients registered at Tripoli Medical Centre were studied in five years period from January 2005 to October 2009. The information were reviewed and the data were collected. A sample from each patient (785 male; 455 female) was analysed for genotyping and sub-typing using specific genotyping assay. The information was correlated with the risk factors studied and the statistical data were analyzed using SPSS version 11.5.

**Results:**

Off the total patients studied, four different genotypes were reported, including genotypes 1, 2, 3, and 4. Genotype4 was the commonest (35.7%), followed by genotype1 (32.6%). According to subtypes 28% were unclassified genotype 4, 14.6% were genotype 1b and some patients infected with more than one subtype (2.3% genotype 4c/d, 1% genotype 2a/c). Genotypes 1 was the commonest among males, while genotype 4 among females. According to the risk factors studied, Genotype1 and genotype 4 were found with most of the risk factors. Though they were particularly evident surgical intervention, dental procedures and blood transfusion while genotype 1 was only followed by genotype 3 mainly which mainly associated with certain risk groups such as intravenous drug abusers.

**Conclusion:**

Here in we report on a detailed description of HCV genotype among Libyans. The most common genotype was type 4 followed by genotype 1, other genotypes were also reported at a low rate. The distribution of such genotypes were also variable according to gender and age. The commonly prevalent genotypes found to be attributable to the medical -related transmission of HCV, such as blood, surgery and dental procedures when compared with other risk factors. This however, raises an alarming signal on the major steps to be taken to reduce such infection in Libya

## Background

Hepatitis C virus (HCV) is the major public health problem and it is one of the most important causes of chronic liver diseases all over the world. Studying the epidemiology of such problematic virus plays an important role on the methods of its prevention [1-4]. Such epidemiology varies geographically and temporally due to distribution and evolution of risk factors. In Europe and North America the prevalence of HCV is about 1% [5,6]. Though it was higher among southern Italy and Southern Spain as it varied between 8.4%-22.4%. In North Africa and Arabian countries such prevalence was between 1.4% and 2.1% in certain countries such Libya, Tunis and Saudi Arabia, though it was the highest in Egypt as it reached up to 19.3% [7,8]. Furthermore the epidemiology of HCV was highly associated with a certain risk groups such as: blood and blood products, Intravenous drug abusers (IVDA), sexual transmission, inadequate sterilizing medical equipment, body piercing and sharing razors and other personal items which contaminated with HCV [9].

Hepatitis C virus has been characterized by having a higher rate of spontaneous mutation that leads to a marked degree of heterogeneity among its genotypes [10]. The genus hepacivirus consists of six phylogentically distinct clades (genotypes) from1 to 6 and more than 70 subtypes (termed a, b, c, d,.etc...) of HCV [10,11]. The epidemiology of such genotypes has been to be variable among different geographical regions worldwide. However HCV genotypes 1,2 and 3 are commonly distributed all over the world though 4,5, and 6 were mainly found in a certain areas. HCV genotype 4 particularly prevalent in Northern and Central African counties particularly Egypt, whereas HCV genotype 2 is frequent in West Africans countries. Where genotypes (5) and (6) are common in South Africa and Asia respectively [12]. The heterogeneity of HCV genotypes also plays a role in understanding the epidemiology among different risk population. HCV genotype 3a was found to be associated with drug addicts and genotype 1b in blood transfusion. Furthermore a single subtype was found to be associated with certain areas such as genotype 4a in Egypt and genotype 5a in South Africa [13].

A few studies on the epidemiology of HCV among Libyans were reported. These include the sero-prevalence HCV among different risk groups including health care workers, blood donors, renal dialysis patients and multi blood transfused patients [14], but the HCV genotype distribution remains to be determined. Hence then epidemiological studies of HCV genotypes among Libyans may provide a good understanding on the nature of HCV infection and its spread. The objectives of this study were to determine the epidemiology of HCV genotypes among different Libyan patients and its association with the risk factors involved and how this could be reflected on the prevention of such virus among the Libyan society.

## Methods

### Patient population

A total of 1240 patients with hepatitis C virus were Studied. They were recruited from the Department of Infectious Diseases at Tripoli Medical Centre, Tripoli. The participation was voluntary in accordance of with the guidelines for observational and interventional studies from ethical committees of our National ethical Standards as the research was conducted according to Helsinki Declaration (2000) [15].

The patients were registered and followed up at Out Patient Department from January 2005 to October 2009. Seven hundred and eighty five patients studied were male and 455 patients were female. Their age ranged from16 to 84 years with an average age of 45 years. The study was designed to collect the data and extract information from each patient including age, gender, year of diagnosis and risk factors for HCV such as history of blood transfusion, intravenous drug abuse (IVDA), history of surgical intervention, family history of HCV positive and history of promiscuity and dental procedure. Those patients who denied any risk factors were assigned to be as an; unknown group.

### Patient Selection Criterion

The study was designed to investigate the impact of HCV genotypes on the epidemiological manifestations of HCV infection among Libyan patients. Each patient has to fulfil the following criteria; no co-infection with human immune-deficiency syndrome (AIDS) virus or with hepatitis B virus and Hepatitis D Viruses, and non of them had liver cirrhosis, or undergo haemodialysis.; and no concomitant metabolic or autoimmune disorder or underlying systemic diseases all patients enrolled in this study were restrictedly chosen to fulfil the criteria mentioned.

### Laboratory and Clinical Evaluation of HCV Infection

A serum specimen was collected from each patient and was tested positive for HCV antibody (Anti-HCV) using and 3^rd ^generation commercial Enzyme Linked Immunosorbant Assays (ELISA)[The **INNO-LIA^™ ^HCV Ab III update **(Belgium) is 3^ed ^generation line immunoassay which incorporates HCV antigens derived from the core region, the E2 hypervariable region, the NS3 helicase region, the NS4A, and NS5A regions. The antigens were coated as 6 discrete lines on a nylon strip with plastic backing. In addition, four control lines are coated in each strip: strepavidin control, 3+ Positive Control (anti-human Ig), 1+ Positive Control (human IgG) and ± cutoff line (human IgG). The stirp incubated with test sample then we add purified alkaline phosphatase - labelled goat anti -human IgG last we add conjugate]. Such immunoassay was known to have a high specificity and sensitivity (over 99%), with minimal or no limitation of detecting HCV antibody.

### Determination of HC Viral Genotypes

HCV genotyping was performed by gene amplification using COBAS-Amblicor HCV test { this test detected by reverse-transcribing HCV RNA into cDNA by PCR, hybridizing amplified cDNA with an oligonucleotid probe that binds enzyme, and catalyzing conversion of substrate to a colored product that is recognized by COBAS AMBLICOR Analyzer (Roche, Diagnostic, Basal, Switzerland). Such analysis is in worldwide use and it covers all the six internationally recognized HCV genotypes.

### Statistical Analysis

Quantitative variables were expressed as mean ± standard deviation (X ± SD)and were compared by Student's test (t-test). Differences in proportion of qualitative variables were tested with non-parametric tests (X_2_) Yates correlation. Fisher exact test and a *p *value < 0.05 were considered significant. A multivariate analysis was conducted using logistic regression in order to verify which variables statistically had an influence on HCV infection. such as gender (male vs. female), IV drug abuser (yes or no), blood transfusion (yes or no) surgical Intervention and blood transfusion (yes or no), dental care (yes or no); promiscuity (yes or no). The data were analyzed using SPSS version 11.5 to identify the distribution of different genotypes and its association with gender, age, year of diagnosis and risk factors.

## Results

### Prevalence of HCV genotypes among the populations studied

A total of 1240 patients were studied during a five year period from 2005to 2009 as shown in Table 1. Off these patients, 785 (63.3%) were predominantly males and 455 (36.7%) patients were females with a male; female ratio (1.7:1). The age was ranged from 16 to 84 years with a predominantly larger proportion of younger patients with an average of 40 years or less with no significant gender variation (*P *> 0, 05). The prevalence of HCV in this study was calculated, per year and expressed as a percentage.

**Table 1:** Prevalence of HCV genotypes among the patient studied

Gender			
**Genotype/subtype**	**Male (%)**	**Female (%)**	**Total (%)**

Genotype 1	270(34.3)	134(29.5)	404(32.6)

1a	42 (5.3)	18 (3.9)	60 (4.9)

1b	114 (14.5)	67 (14.8)	181 (14.6)

1(UC*)	114 (14.5)	49 (10.8)	163 (13.2)

Genotype 2	108(13.9)	78(17.1)	163 (13.2)

2a	2(0.3)	3 (0.6)	5(0.4)

2b	2 (0.3)	3 (0.6)	5 (0.4)

2c	2 (0.3)	8 (1.7)	10 (0.8)

2a/c	5 (0.6)	8 (1.7)	13 (1)

2(UC*)	97 (12.2)	56 (12.5)	153 (12.3)

Genotype 3	171(21.8)	36(7.9)	207(16.7)

3a	24 (3)	5(1.1)	29 (2.3)

3(UC*)	147 (18.8)	31 (6.8)	178 (14.4)

Genotype 4	207(30)	236(45.5)	443(35.7)

4a	34 (4.3)	34 (7.4)	68 (5.4)

4c/d	23 (2.9)	5 (1.1)	28 (2.3)

4(UC*)	179 (22.8)	168 (37)	347 (28)

Total (%)	785 (100)	455 (100)	1240 (100)

*UC; *unclassified viral genotype*

Different HCV genotypes were found among the Patients studied as shown in Table 1. These include genotypes, 1,2,3 and 4 while HCV genotype 5 & 6 were not reported among these patients. The prevalence of such genotypes was variable among the patients, Genotype 4 was the most frequent one detected in 443 (35.7%)patients followed by genotype 1 in 404 (32.6%) patients, and then genotypes 3 and 2 accounted for 207(16.7%) and 186 (15%) patients respectively. The year by year distribution has been stable since 2005; the beginning of the study including the high percentage of type 4 and 1.

The prevalence of gender associated HCV genotypes was analysed among the patients. The most frequent genotype reported among females was genotype 4 as it was accounted for 207(45.5%) patients, followed by genotype 1 accounted for 134 (29.5%), G2 accounted 78 (17.1%) and then genotype 3 accounted for 36 (7.9%). The most frequent HCV genotype among male populations was genotype 1 as it was detected in 270(34.3%) cases, followed by genotype 4 detected in 236 (30%) cases, genotype 3 detected in 171 (21.8%) and less frequent one was HCV genotype 2 detected in 108 (13.9%). The relationship between HCV genotype and gender was statistically significant (*P *value = 0.00).

Different sub-genotypes were reported among the patients studied. Genotype 1 b was among the frequent as it accounted for 181(14.6%) without no gender variation, followed by genotype 4a 68(5.4%) most predominantly among females 34 (7.4%). Then genotype 1a accounted for 60 (4.9%) patients in 42 (5.3%) males and 18(3.9%) females. HCV genotype 3a accounted for 29(2.3%) patients male 24(3%) female 5(1.1%). Genotype 2 was The most heterogenic genotype as four different subtypes were reported which include genotype 2a, 2b, 2c & 2a/c.

### Distribution of HCV genotypes according to age

The prevalence of HCV genotypes among Libyan population is shown in Table 2. There was a variable distribution of the genotype according to the age group. Genotype 1 was associated with a younger age group between 15-34, decreased in a middle-aged group 35-44 and was less at age more than 55 years (45.1%, 33.2%, 8.9%, 12.8%). Conversely, HCV genotype 2 was higher among older age above 55 years, less at age group 45-54, and 35-44 and lesser at a younger age 15-34 years (13.4%, 18.3%, 22.6%, 45.7%). Genotypes 3 & 4 were most associated with patients aged less than 45 years genotype 3; 88.4% where it was 11.6% for patients aged more than 44 years, and genotype 4; 69.8%in patient less than 45 years old and it counted 30.2% for patients aged more than 44 years (P = 0.00).

**Table 2:** Prevalence of HCV genotypes among different age groups

Age (Years)				
Genotype/subtype(%)	15-34	35-44	45-54	55-85

Genotype 1a	34(56.7)	10(16.7)	5(8.3)	11(18.3)

1b	65(35.7)	67(37.2)	21(11.4)	28(15.7)

1 (UC*)	83(50.8)	57(34.9)	10(6.4)	13(7.9)

Genotype 2a	0	0	0	5(100)

2b	0	0	0	5(100)

2c	2(20)	6(60)	0	2(20)

2a/c	2(15.4)	0	3(23.1)	8(61.5)

2 (UC*)	21(13.6)	28(18.6)	39(25.4)	65(42.4)

Genotype 3a	21(72.7)	8(27.3)	0	0

3 (UC*)	90(50.7)	64(36.2)	13(7.3)	11(5.8)

Genotype 4a	21(30.8)	18(27)	13(19.2)	16(23)

4c/d	12(45.4)	10(36.4)	3(9.1)	3(9.1)

4 (UC*)	121(35)	127(36.6)	36(10.4)	63(18)

Total(%)	472(38.1)	395(31.9)	143(11.5)	230(18.5)

*UC; *unclassified viral genotype*

### Association of the genotypes with the risk factors

The risk factors associated with the transmission of HCV were determined in all patients studied as shown in Table 3. The most frequently reported risk factor was a history of surgical procedure accounted for 300 patients (24.2%), (176 (58.6%) males and 124 (41.4%) females. Followed by patients with history of blood transfusion 212 (17.1%), (109(51.2%) males and 103 (48.8%) females. Intravenous drug abuser 'IVDA' reported 85 (6.9%) patients (all males). Patients who had history of dental procedures were 225 (18.2%) patients, 142 (63.2%) males, 83 (36.8%) females. Family history of HCV infection was recorded in 80(6.5%), patients 64(80%) males and 16(20%) were females. History of promiscuity were recorded in 27(2.1%), patients all of them were males. The patients denied any history of risk factors were recorded in 311 (25.1%), 181 (58.3%) males and 130 (41.7%) females. The relationship between HCV risk factor for infection and gender was statistically significant 'P value = 0.000'.

**Table 3:** Prevalence of genotypes according to risk factors studied

Risk factors (No Patients)							
**Genotype/subtype**	**Blood Transfusion**	**IVDA**	**Surgical procedure**	**History of promiscuity**	**Family history of HCV**	**Dental procedure**	**Unknown**

Genotype 1a	18	13	5	0	3	10	11

1b	36	13	39	10	10	34	39

1(UC*)	16	13	41	3	15	31	44

Genotype 2a	0	0	2	0	3	0	0

2b	0	0	5	0	0	0	0

2c	5	0	3	0	0	2	0

2a/c	0	0	8	0	0	3	2

2(UC*)	23	0	52	3	8	34	33

Genotype 3a	8	2	3	3	0	8	5

3 (UC*)	21	23	44	0	10	28	52

Genotype 4a	18	0	16	0	5	8	21

4c/d	10	0	2	0	3	5	8

4 (UC*)	57	21	80	8	23	62	96

Total	212	85	300	27	80	225	311

*UC; *unclassified viral genotype*

Genotype1 and genotype 4 were predominantly associated with most of the risk factors studied, particularly those of previous surgical or dental procedures, blood transfusion and family history. Though, genotype 1 was only followed by genotype 3 in patients with IVDA. The association of between the risk factors and HCV genotypes was found to be statistically insignificant (*P *value = 0.180).

## Discussion

The epidemiological studies on Hepatitis C Virus genotypes have gained major attention all over the world as they appear to play an important role in elucidating the clinical status of such infection. They have shown to be of great benefit in guiding therapeutic decision and implementing proper preventive strategies. The epidemiological patterns of HCV vary greatly among the different countries and even among the regions of the same country. However, little is known about the epidemiology of HCV genotypes in Libya. Hence then carrying such study will provide great understanding on the prevalence of various genotypes among the Libyans which should guide the therapeutic and prognostic implications in HCV infection. In this study the patterns of HCV genotypes and various risk factors for possible route of transmission in Libya were studied. The serum samples collected from different patients registered at Tripoli Medical centre were found to be positive for HCV and could thus be genotyped. Four different genotypes were reported in this study including genotypes, 1,2,3 and 4. The distribution of these genotypes were variable among the patients studied. The most prevalent genotype was genotype 4 and then genotype 2, though genotypes 3&2 were less accounted. Different subtypes were found among HCV genotypes studied. These include nine HCV subtypes as genotype 1 subtype (1a & 1b), 2(2a,2b,2c&2a/c), 3(3a), 4(4a&4c/d) the most frequent of these subtypes were 4a and 1b. The data of the present study is in concordance with previous studies reported from different regions of the world particularly in North African countries. Previous study conducted in Tunisia reported that genotype 1b was the most prevalent genotype [16] and in Egypt genotype 4a was predominant [17], genotypes 1a and 1b were common in the United State and Europe [18,19]. In Pakistan and Japan Genotypes 3a and 1b were common respectively where as genotype 5a were common in South Africa [9]. The distribution such HCV genotypes among Libyan patients remain invariable during the five years study period including the high rate of genotype 4 and 1. This concise with Henquell, *etal*. who found that there was no year to year variation of HCV genotypes in Central France in six years prospectively conducted study [20]. It is apparent that further studies are needed to clarify such evident prevalence as longer time may be needed to observe such changes. In Pakistan it seems that 15-20 years needed as the genotype 3 a to be replaced by genotype 1(a or b) [9], while in Venezuela that took only 10 years time for displacement of genotype 1 b by type 2 [21].

Studies on the association of gender with specific HCV genotypes were found to be equivocal. In Luxembourg the prevalence of HCV genotype 3 was found to be a significantly associated with males while genotype 2 and 5 more frequent in females [22]. In Pakistan such association was lacking as the distribution of HCV genotypes were similar in both male and female patients [23,24]. In this study there was a variation of HCV genotypes among male and female patients. Genotype 4 was significantly more frequent in female while genotype 1 more frequent in males though the frequency of genotypes 3 and 4 were independent of gender. However, such association merits, further investigation.

The distribution of HCV genotypes may be variable according to the age of the population. In Italy, the prevalence of genotypes 1b and 2 decreased significantly among younger children compared with the older ones who have an increased rate of genotypes 3 and 4 [25,26]. In France type 5 was more frequent in patients older than 50 than those younger than 49 [27,28]. Studies carried by Roman *etal*., showed that genotype 3 was associated with patients aged less than 40 years, while genotype 2 was significantly prevalent with those over 40 years of age [22]. In this study, genotype 1 was associated with a younger age group less than 34 years, decreased in a middle- and older age groups. Conversely, HCV genotype 2 was higher among elderly patient above 55 years, lesser at age group of 45-54, and even after. These finding however, do need further investigations, as such epidemiological variation may be associated with the mode of transmission of HCV and thus they may have clinical and therapeutic implications.

Different studies have shown the dynamicity of HCV genotypes, which lead to the emergence of different types and subtypes over time. Such phenomenon is obvious in certain European counties such as in Germany and Greece, where the prevalence of HCV genotypes 1b and 2 have decreased and genotypes 1a, 3a, and 4 increased. Such changes were not noticeable in Luxembourg where genotypes 1 and 3 were the most prevalent [22]. Here in we did not notice any significant changes in the emergence of HCV genotypes among the Libyans during the period of this study. Such results were in agreement with the data reported in Tunis and Egypt [16,17] as HCV genotypes proportion has been relatively constant overtime. The association of HCV genotypes variation with demographic data may be related to the immigration flow which obvious among the European countries and rarely seen in the Arab and African countries.

Many studies have been suggesting that HCV genotypes are associated with certain risk factors and different modes of transmission. The genotypes reported in this study were isolated from all the patients with the risk factors stated. Such, relationship between HCV genotypes and risk factors was statistically significant. High prevalence of HCV genotype 4 in our study is particularly attributable to previous history of surgical operation followed by dental intervention and blood transfusion respectively. The same is applicable for genotype 1. While genotype 3 and 1 were common inpatient with IVDA. Configuration of HCV subtypes and its association with the risk factors was also obvious in this study. HCV genotype 1b is more common in patients who had history of blood transfusion and surgical procedures.

The association of HCV genotypes with the risk factor varies from one country to another. The high prevalence of HCV genotype 3 among Europeans is attributed to IVDA and incarcerated population [28]. In this study only 6.9% of individuals admitted they have a history of IVDA which was associated with genotype3. The high prevalence of genotype 4 and 1 among Libyans which is uncommon in Europe and North America is particularly attributable to medical-related transmission such as blood transfusion, surgery and dental procedures. This is an agreement with studies from Pakistan and Hungry where the majority of cases of genotypes 1 and 4 have history of hospitalization for surgery, dental procedure and blood transfusion [9,23,29]. This raises an important question regarding the prevention methods of to be established among Libyan hospitals to prevent the spread of HCV and other blood born viruses [30,31].

This study showed a detailed estimation on the epidemiology of HCV genotypes in Libya. The commonly prevalent genotypes 4 and 1 are more likely attributable to the medical-related transmission such as blood, surgery and dental procedures despite that blood banks in Libya do screen routinely for HCV in all blood products. This however raises an alarming signal on the major steps to be taken to reduce such infection. Therefore, a national strategy should be implemented and specific prevention guide lines have to be followed to reduce such risks. As Libya being a third largest country in Africa with a variable race populations further studies are needed in different regional parts of the country to estimate the diversity of HCV genotypes in each region and assess the risk factors involved with specific emphases on the genetic sequences of un-typable HCV genotypes and multivariate analysis on such risk factors [32].

## Conclusion

The predominantly isolated HCV genotype from Libyans were genotype 4 followed by genotype 1. Other genotypes and subtypes such as 1(a,b,c),2(a,b,c),3a, and 4(a,c/d) were also reported in this study. Such genotypes were variable according to the age and gender of the patients studied. The mostly associated risk factors with these genotypes were medical-related route of transmission including blood transfusion, surgical operation and dental procedures, other risk factors such as family history and IVDA were also reported.

## Competing interests

The authors declare that they have no competing interests.

## Authors' contributions

All authors have read and approved the final manuscript and contributed immensely in the study. HAE; Conceived the study, collected the epidemiological data and analyzed data statistically and helped in writing and revising the manuscript. YMA; Helped in the study, analysing and revising the data. BAA; Treating and following up patients, helped in designing and writing the manuscript. MAD; Designed and supervised the study, writing and revising the manuscript; a leading expert in Nosocomial infections and Microbial Epidemiology
